# Evolutionary history of endangered and relict tree species *Dipteronia sinensis* in response to geological and climatic events in the Qinling Mountains and adjacent areas

**DOI:** 10.1002/ece3.6996

**Published:** 2020-11-11

**Authors:** Khurram Shahzad, Mi‐Li Liu, Yu‐He Zhao, Ting‐Ting Zhang, Jian‐Ni Liu, Zhong‐Hu Li

**Affiliations:** ^1^ Key Laboratory of Resource Biology and Biotechnology in Western China Ministry of Education College of Life Sciences Northwest University Xi'an China; ^2^ Department of Geology Early Life Institute State Key Laboratory of Continental Dynamics Northwest University Xi'an China

**Keywords:** climatic oscillation, conservation, *Dipteronia sinensis*, lineage divergence, mountain uplift, population structure

## Abstract

Geological and climatic events are considered to profoundly affect the evolution and lineage divergence of plant species. However, the evolutionary histories of tree species that have responded to past geological and climate oscillations in central China's mountainous areas remain mostly unknown. In this study, we assessed the evolutionary history of the endangered and relict tree species *Dipteronia sinensis* in the Qinling Mountains (QM) and adjacent areas in East Asia based on variations in the complete chloroplast genomes (cpDNA) and reduced‐genomic scale single nucleotide polymorphisms (SNPs). Population structure and phylogenetic analysis based on the cpDNA variations suggested that *D. sinensis* could be divided into two intraspecific genetic lineages in the eastern and western sides of the QM (EQM and WQM, respectively) in East Asia. Molecular dating suggested that the intraspecific divergence of *D. sinensis* occurred approximately 39.2 million years ago during the later Paleogene. It was significantly correlated with the orogeny of the QM, where the formation of this significant geographic barrier in the region may have led to the divergence of independent lineages. Bayesian clustering and demographic analysis showed that intraspecific gene flow was restricted between the EQM and WQM lineages. Isolation‐with‐migration analysis indicated that the two genetic lineages experienced significant demographic expansions after the Pleistocene ice ages. However, the genetic admixture was determined in some populations between the two lineages by the large scale of SNP variations due to DNA incompatibility, the large significant population size, and rapid gene flow of nuclear DNA markers. Our results suggest that two different conservation and management units should be constructed for *D. sinensis* in the EQM and WQM areas. These findings provide novel insights into the unprecedented effects of tectonic changes and climatic oscillations on lineage divergence and plant population evolution in the QM and adjacent areas in East Asia.

## Graphical abstract

This novel study describes the unprecedented details of geological and climatic changes profoundly shaped the lineage divergence and population evolution of *Dipteronia sinensis* plant species in the Qinling Mountains in central China. The readers will take help to understand the mechanism of lineage divergence due to the mountain's orogenesis and repeated climatic changes.

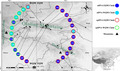

## 1 INTRODUCTION

Geological events and repeated climate oscillations have affected the genetic architecture of plant species and reshaped the divergence of their lineages (Abbott et al., 2000; Avise, 2000; Hewitt, 2000; Hickerson et al., 2009; Liu et al., 2012; Wen et al., 2014). Previously, it was shown that tectonic uplift could change the environmental conditions to stimulate species divergence and produce novel species lineages and/or adaptive zones. Factors such as mountain uplift and climatic cycles can alter the genetic compositions by limiting gene flow to affect evolutionary processes and result in lineage divergence in mountain populations (Avise, 2000; Hewitt, 2001; Hickerson et al., 2009). However, montane orogenesis led to fluctuations in the climate in high‐altitude regions during the Quaternary period to severely impact the vegetation biota and genetic architecture by preventing population expansion and the migration of species with narrow thermal tolerance ranges (Avise, 2000; Knowles, 2001). Previous highly cited studies broadly explored the effects of tectonic uplifts and climatic oscillations on the diversification patterns of plants species in Europe, North America, and other regions, for example, in the Sierra Madre of Mexico (Bryson et al., 2012), the Andes (Hughes & Eastwood, 2006), Costa Rica (Muñoz et al., 2019), and the Himalayas (Xu et al., 2010; Yang et al., 2012). In China, many previous studies focused on this phenomenon in the complex and conspicuous topographical regions on the Qinghai–Tibet Plateau and adjacent areas (Ding et al., 2020; Wen et al., 2014; Xing & Ree, 2017). It was recently reported that climate factors and montane orogenesis had strong and direct effects on montane forests, and they shaped the montane plant diversity (Li et al., 2020; Muñoz et al., 2019). However, another critical biodiversity hotspot in central China is the Qinling Mountains (QM), and the detailed evolutionary histories of plant species in this area are generally unknown (Liu et al., 2015; Wan et al., 2016; Zhang et al., 2015).

The QM comprises a unique range of mountains that run in an east–west direction in China's center, which provide large‐scale complex and heterogeneous habitats (Jia et al., 2010; Qin et al., 2014). The QM is also an important geographical and biodiversity hotspot in Eastern Asia, where it contains more than 337 endangered Chinese plant species. The original development of the QM landforms was formed by the collision between the Yangtze block and north China block during the Mesozoic and Cenozoic periods (Zhang et al., 1996), and the subsequent lateral extrusion of the Tibet Plateau in the early Cenozoic (approximately 40 Mya). In addition, the present high topographic gradients with a height drop of 1,000–2,000 m in the QM and the highest peak, Taibai Mountain, which is 3,767 m, resulted from the latest rapid uplift of the Kun‐Huang tectonic changes and Gonghe mountains uplift activated by the Tibetan Plateau during the late Miocene and early Pleistocene (An et al., 2001; Dong et al., 2011; Ying, 1994; Zhang et al., 2006). Moreover, some high mountains in the Qinling region (e.g., Taibai Mountain) were probably covered by ice caps during the Pleistocene ice ages (Li et al., 2004; Rost, 1994). In combination with complex tectonic changes and harsh climatic oscillations, these mountains influenced the evolutionary history of various organisms (Bennett, 2004; Hua & Wiens, 2013; Zink et al., 2004). However, the populations present on high mountains that expanded during the postglacial periods might have contracted into low elevation refugia during the Pleistocene ice ages.

Moreover, several endemic species inhabit this region, which supports distinct habitats and climatic oscillations on the slopes of both temperate and subtropical ecosystems in the QM area that serve as the boundary of the Palearctic and Oriental realms between north and south China (Chen, 2008; Zhang, 2004). However, the QM could serve as a significant barrier to generate the north–south break for species generally distributed in the low altitude localities of the northern and southern sides, for example, <1,200 m above sea level (Qu et al., 2009; Yan et al., 2010). Some species in the high‐altitude localities of the QM were also impacted by Pleistocene climatic fluctuations, which resulted in their diversification in the west–east and west–central–east breaks (Fang et al., 2015; Wang et al., 2013; Yang et al., 2015). A recent study reported three lineages in the QM, that is, a northwestern QM lineage comprising the Tibet lineage, as well as eastern and western lineages, which separated about 3–4 million years ago (Mya) (Huang et al., 2017). However, our understanding of how geological events and climatic changes during the Pleistocene might be associated with spatial distributions, lineage sorting, and population evolution is restricted due to a lack of experimental observations (Li et al., 2009; Wang et al., 2012) in this important region of the QM with high biodiversity.

*Dipteronia sinensis* (Sapindaceae) is an endangered and relict woody plant species with ecological and economic value, mainly distributed in the QM and adjacent areas (Acevedo‐Rodríguez et al., 2011). This species provides a good model for investigating the effects of geological and climatic events on intraspecific divergence and population evolution. Previous molecular phylogenetic studies demonstrated that *Dipteronia* and *Acer* are sister genera based on variations in their complete chloroplast genomes (cpDNA) (Renner et al., 2008; Yang et al., 2010) and single gene experiments based on cpDNA/nuclear DNA (Gao et al., 2017). However, other studies based on nuclear ribosomal DNA, cpDNA, and ITS/cpDNA showed that the phylogenetic relationship between *Dipteronia* and *Acer* is more distant, where they formed paraphyletic groups (Yang et al., 2010; Zhou et al., 2016). Moreover, *Dipteronia* populations may have experienced a genetic bottleneck (Yang et al., 2008) that lasted over a million years. However, the evolutionary history and divergence during the glacial and postglacial periods are not well understood due to data limitations and the lack of management and conservation for this tree species. Therefore, in this study, we analyzed the cpDNA and nuclear DNA for *D. sinensis* to determine the diversification, population genetic structure, and lineage divergence in this tree species under the impacts of mountain orogenesis and climatic oscillations in the QM and adjacent regions. In particular, we aimed: (a) to investigate how the divergence of the *D. sinensis* lineages might have been related to the orogenesis of the QM and past climatic oscillations; (b) to determine whether gene flow among populations was limited due to the geographical barrier and climate oscillations; and (c) to identify suitable conservation and management strategies to maintain the genetic variation in this endangered tree species.

## 2 MATERIALS AND METHODS

### 2.1 Plant samples and DNA extraction

We sampled 25 populations of *Dipteronia sinensis* (one individual from each population) from widespread localities in Sichuan, Hubei, Henan, Gansu, and Shaanxi provinces in its distribution range in the QM and adjacent regions of China. Detailed information for all of the sampled populations is provided in Table 1. All materials and documents were deposited in the College of Life Sciences, Northwest University, Xi'an, China. Genomic DNA was isolated using 5 mg of dried leaves from a sample of each *D. sinensis* population (preserved in silica gel) before DNA extraction with the CTAB method (Doyle, 1987) or a Tiangen plant DNA extraction kit (Beijing, China). The DNA quality was checked with a 1% agarose gel based on the high molecular weight bands after gel electrophoresis.

**TABLE 1:** Distribution and geographic information (longitude, latitude, and location) for the *Dipteronia sinensis* population samples

No.	Population	Longitude (°)	Latitude (°)	Location
1	MJS	106	34.36	Mt. Maiji, Gansu
2	DC	106.14	34.34	Dangchuan, Gansu
3	LSH	106.01	34.28	Lengshuihe, Gansu
4	DJ	105.94	34.25	Dujia, Gansu
5	LWG	106.13	34.31	Longwanggou, Gansu
6	YB	105.7	33.03	Yangba, Gansu
7	LD	106.47	33.7	Liangdang, Gansu
8	LY	106.29	33.6	Lveyang, Shaanxi
9	ZBS	106.77	33.7	Mt. Zibai, Shaanxi
10	DB	106.85	33.66	Dabagou, Shaanxi
11	FP	107.98	33.68	Foping, Shaanxi
12	NS	108.29	33.65	Ningshan, Shaanxi
13	HHG	107.74	34.11	Honghegu, Shaanxi
14	TBS	107.99	34.26	Mt. Taibai, Shaanxi
15	TY	108.35	33.9	Tianyu, Shaanxi
16	XH	108.42	33.84	Xihe, Shaanxi
17	ZA	108.65	33.44	Zhenan, Shaanxi
18	NBL	109.58	33.52	Niubeiliang, Shaanxi
19	XX	111.76	33.63	Xixia, Henan
20	AK	109.27	32.01	Ankang, Shaanxi
21	WJG	110.55	31.4	Wanjiagou, Hubei
22	LMH	110.49	31.32	Longmenhe, Hubei
23	XE	109.67	30.04	Xuanen, Hubei
24	HH	110.54	30.08	Houhe, Hubei
25	HPS	110.54	30.03	Mt. Huping, Hunan

### 2.2 Assembly and annotation of cpDNA

High‐quality DNA was sequenced using a 350‐bp insert fragment by Novogene Company (Beijing, China). We then used the default parameters in NGSQC Toolkit v 2.3.3 to extract the clean data (Patel & Jain, 2012). After trimming, the clean reads were assembled using MITObim v 1.8 with GenBank Accession No. NC_029338 as the reference sequence (Zhou et al., 2017). Finally, the consensus sequences for each population data set were aligned and visually inspected in Geneious software V.7.1.5 and compared with a previously published *Dipteronia sinensis* genome (NC_029338) (Zhou et al., 2017). The newly sequenced complete chloroplast genomes were deposited in the NCBI GenBank under accession numbers MK193760–MK193784.

### 2.3 Single nucleotide polymorphism (SNP) detection

In order to detect SNPs in nuclear DNA, 23 qualitative genomic DNA samples were selected from 23 populations (one sample from each population). We used quantitative real time‐PCR to assess the quality of the DNA fragments before nuclear DNA library construction. Restriction endonuclease enzymes were applied to amplify each genome sample. All of the samples were mixed after PCR before selecting good quality fragments with the desired length based on the results of capture arrays, which were then used to construct the DNA library. The genotyping‐by‐sequencing technique was employed to sequence the selected nDNA library fragments. After sequencing, the raw reads were filtered based on the paired‐end reads.

High‐throughput sequencing obtained 23.15 GB of raw reads data from 23 samples, with an average of 0.99 GB per sample. The clean reads comprised 23.14 GB and the average amount per sample was 0.99 GB. After high‐quality sequencing (Q20 ≥ 92.8%, Q30 ≥ 82.85%), we also obtained the GC contents of the 23 *Dipteronia sinensis* samples. Burrows–Wheeler aligner software was used to align the clean data (Li & Durbin, 2009), and the DJ population was used as the reference genome. High‐quality SNP loci were filtered with SAMtools and used for subsequent analyses (Li et al., 2009). GENALEX V.6.5 (Peakall & Smouse, 2012) was employed to detect the physical linkages between any pairs of loci.

### 2.4 Phylogenetic analysis

Gene genealogies based on complete chloroplast genomes (cpDNA) were reconstructed by using MEGA v 7.0 and MrBayes v 3.2.3 (Ronquist & Huelsenbeck, 2003) to obtain the maximum likelihood (ML), maximum parsimony (MP), and Bayesian inference (BI) cladograms. To examine the phylogenetic relationships among the 25 populations of *Dipteronia sinensis*, we used the complete chloroplast genomes for *Acer buergerianum* (NC_034744.1), *A. morrisonense* (NC_029371.1), *A. miaotaiense* (NC_030343.1), *A. griseum* (NC_034346.1), *A. palmatum* (NC_034932.1), *A. truncatum* (NC_037211.1), *Sapindus mukorossi* (NC_025554.1), *D. dyeriana* (NC_031899.1), *Litchi chinensis* (NC_035238.1), *Dodonaea viscosa* (NC_036099.1), *Koelreuteria paniculata* (NC_037176.1), and *Dimocarpus longan* (NC_037447.1) as outgroup taxa. jModelTest and PAUP* v 4.0 software were used to obtain the general time‐reversible model and gamma (G) distribution for the rate variation among sites based on Akaike's information criterion (Swofford, 2002). For the ML and MP phylogenetic cladogram analyses, we conducted 1,000 bootstrap replicates. To construct the best phylogenetic tree in MrBayes, we kept the burn‐in as 2,500 and retained every 1,000 generations from 30,000,000 random tree rotations. The FigTree program was used to visualize the output (Rambaut, 2009).

We used 23 *Dipteronia sinensis* populations to perform phylogenetic analysis with high‐quality nDNA SNP data. TreeBest software (http://treesoft.sourceforge.net/treebest.shtml) was used to construct a neighbor‐joining phylogenetic tree based on 1,000 bootstrap values.

### 2.5 Estimation of divergence time (cpDNA)

The BI method in BEAST v 1.8.4 was used to estimate the divergence times for the intraspecific *Dipteronia sinensis* lineages (Drummond & Rambaut, 2007). We used a fossil age of 56 Mya as the calibration point for divergence between *Dipteronia* and *Acer* (Feng et al., 2018). We modeled two most recent coalescent ancestral (MRCA) points to obtain the best divergence time results (Guo et al., 2014) by using the log‐normal distribution of the first calibration point with mean = 0, *SD* = 1, and offset = 60 Mya for the complete chloroplast genome data sets including outgroups. The crown age for the sister genera *Acer* and *Dipteronia* was used as one calibration point with a normal distribution, mean = 56 Mya, and *SD* = 1.

The Yule speciation tree prior and an “uncorrelated relaxed clock” model were employed with a normal prior distribution for the branch lengths. We set 50,000,000 Markov chain Monte Carlo (MCMC) algorithm steps, with 5,000,000 steps as the burn‐in, and collected the parameters after every 5,000 steps. We used Tracer v 1.5 software (Rambaut & Drummond, 2009) to summarize the final tree by selecting the median heights and maximum clade credibility to check the MCMC chain's convergence. TreeAnnotator v 2.4.5 was used to remove 10% of the total trees. FigTree v 1.3.1 was used to obtain summary statistics and visualize the maximum clade credibility tree (Rambaut, 2009).

### 2.6 STRUCTURE analysis

The model‐based Bayesian algorithm in STRUCTURE v 2.3.4 software was used to analyze the genetic structure of *Dipteronia sinensis*. We implemented an admixture model with the additional correlated allele frequency between populations, as recommended in a previous study (Falush et al., 2007). Unlinked SNPs (one SNP site per locus) were utilized based on the whole chloroplast genome. The outgroup individuals were not included in this analysis. In STRUCTURE, 200,000 MCMC cycles were conducted after 100,000 burn‐in cycles. Eight simulations were conducted to obtain clusters (K) from 1 to 10.

STRUCTURE analysis based on nDNA SNP data was conducted to determine the genetic variation in a non‐random distribution of a species or population. According to the geographical distribution, a population or group of populations can be divided into several sub‐groups. Different individuals with strong genetic relationships lie closer and within the same group. By contrast, the populations with weak genetic relationships are located at greater distances. Population structure analysis is useful for understanding the evolutionary process and for associated studies of genotypes and phenotypes. The online program PLINK (http://pngu.mgh.harvard.edu/~purcell/plink/) was used for STRUCTURE analysis based on 23 *Dipteronia sinensis* populations. A PLINK input file (‐Ped file) was prepared to construct the population genetic structure and lineage information. The DJ population was used as the reference genome.

### 2.7 Population dynamics history

To analyze the coalescent‐based isolation‐with‐migration (IM) model for *Dipteronia sinensis*, we implemented six parameters in IMa software (Hey, 2009). All of the parameters were scaled by the neutral mutation rate by setting (as an autosomal example): q1 = 30, q2 = 10, qA = 50, t = 30, m1 = 10, and m2 = 10. The IM model determines the divergence time “t” in the form of generations between two related populations. We conducted 5,000,000 additional steps with a burn‐in of 500,000 steps to evaluate the surfaces/posterior distribution likelihoods. Based on the whole chloroplast genomes for 25 populations of *D. sinensis*, 252 SNP loci were divided into two groups according to the phylogenetic analysis (the east clade group contained 15 populations and the west clade group contained 10 populations) to analyze the demographic history and divergence time (generations). The genetic diversity parameters were also investigated for each lineage by using DnaSP v 5.0, including the nucleotide diversity Pi (*π*) and theta (*θ*) (Nei & Li, 1979).

Principal component analysis (PCA) was conducted based on nDNA SNP data to visualize the significant axes of variation in the population genetics using the adegenet package (Jombart, 2008) in R software. We conducted PCA using the following formula.

$$d_{\mathrm{ik}}^{\prime }=\frac{\left(d_{\mathrm{ik}}‐E\left(d_{k}\right)\right)}{\sqrt{E\left(d_{k}\right)\times \left(1‐E\left(d_{k}\right)/2\right)/2}}$$

PCA calculates the number of individuals “*n*” = XX autosomal data, while ignoring more than two alleles and inconsistent data. In the equation above, *“i*” is the number of individuals, “*k*” is the position of an SNP, *d_ik_* indicates that if an individual “*i*” and the reference allele are homozygous, then *d_ik_* = 0, whereas if they are heterozygous, then *d_ik_* = 1, and if the “*i*” individual is homozygous for the non‐reference allele, then *d_ik_* = 2. In this study, the eigenvectors and eigenvalues were calculated using GCTA (http://cnsgenomics.com/software/gcta/pca.html) software and the PCA plot was drawn using R software.

## 3 RESULTS

### 3.1 Lineage relationships and divergence

The cpDNA data set comprised 25 population samples, where each genome had similar a typical quadripartite structure to those of most land plants. The newly sequenced chloroplast genomes were identical to the previously published plastomes for *Dipteronia sinensis* in terms of their structure, tRNAs, mRNAs, and gene contents (Table 2) (Zhou et al., 2017). The chloroplast genome data set for each population had different assembly reads and aligned base‐pair length ranges. The numbers of tRNAs, mRNA, and protein‐coding genes were the same as those in the previously published genome (Table 2).

**TABLE 2:** List of all the chloroplast genes determined in *Dipteronia sinensis* in this study

Gene group	Gene name				
Ribosomal RNA gene	*rrn4.5*	*rrn5*	*rrn16*	*rrn23*	
Transfer RNA gene	*trnA‐UGC*	*trnC‐GCA*	*trnD‐GUC*	*trnE‐UUC*	*trnF‐GAA*
	*trnG‐GCC*	*trnG‐UCC*	*trnH‐GUG*	*trnI‐CAU*	*trnI‐GAU*
	*trnK‐UUU*	*trnL‐CAA*	*trnL‐UAA*	*trnL‐UAG*	*trnfM‐CAU*
	*trnM‐CAU*	*trnN‐GUU*	*trnP‐UGG*	*trnQ‐UUG*	*trnR‐ACG*
	*trnR‐UCU*	*trnS‐GCU*	*trnS‐GGA*	*trnS‐UGA*	*trnT‐GGU*
	*trnT‐UGU*	*trnV‐GAC*	*trnV‐UAC*	*trnW‐CCA*	*trnY‐GUA*
	*trnG*	*trnP‐GGG*			
Small subunit of ribosomes	*rps2*	*rps3*	*rps4*	*rps7*	*rps8*
	*rps11*	*rps12*	*rps14*	*rps15*	*rps16*
	*rps18*	*rps19*			
Large subunit of ribosomes	*rpl2*	*rpl14*	*rpl16*	*rpl20*	*rpl22*
	*rpl23*	*rpl32*	*rpl33*	*rpl36*	
DNA‐dependent RNA polymerase	*rpoA*	*rpoB*	*rpoC1*	*rpoC2*	
Translational initiation factor	*infA*				
Subunit of photosystem I	*psaA*	*psaB*	*psaC*	*psaI*	*psaJ*
	*ycf1*	*ycf2*	*ycf3*	*ycf4*	*Ycf15*
Subunit of photosystem II	*psbA*	*psbB*	*psbC*	*psbD*	*psbE*
	*psbF*	*psbH*	*psbI*	*psbJ*	*psbK*
	*psbL*	*psbM*	*psbN*	*psbT*	*psbZ*
NADH oxidoreductase	*ndhA*	*ndhB*	*ndhC*	*ndhD*	*ndhE*
	*ndhF*	*ndhG*	*ndhH*	*ndhI*	*ndhJ*
	*ndhK*				
Subunits of cytochrome	*petA*	*petB*	*petD*	*petG*	*petL*
	*petN*				
Subunits of ATP synthase	*atpA*	*atpB*	*atpE*	*atpF*	*atpH*
	*atpI*				
Large subunit of RuBisCO maturase	*rbcL*				
	*matk*				
Envelope membrane protein	*cemA*				
Subunit of acetyl‐CoA	*accD*				
C‐type cytochrome synthesis gene	*ccsA*				
ATP‐dependent protease subunit	*clpP*				

**FIGURE 1:**
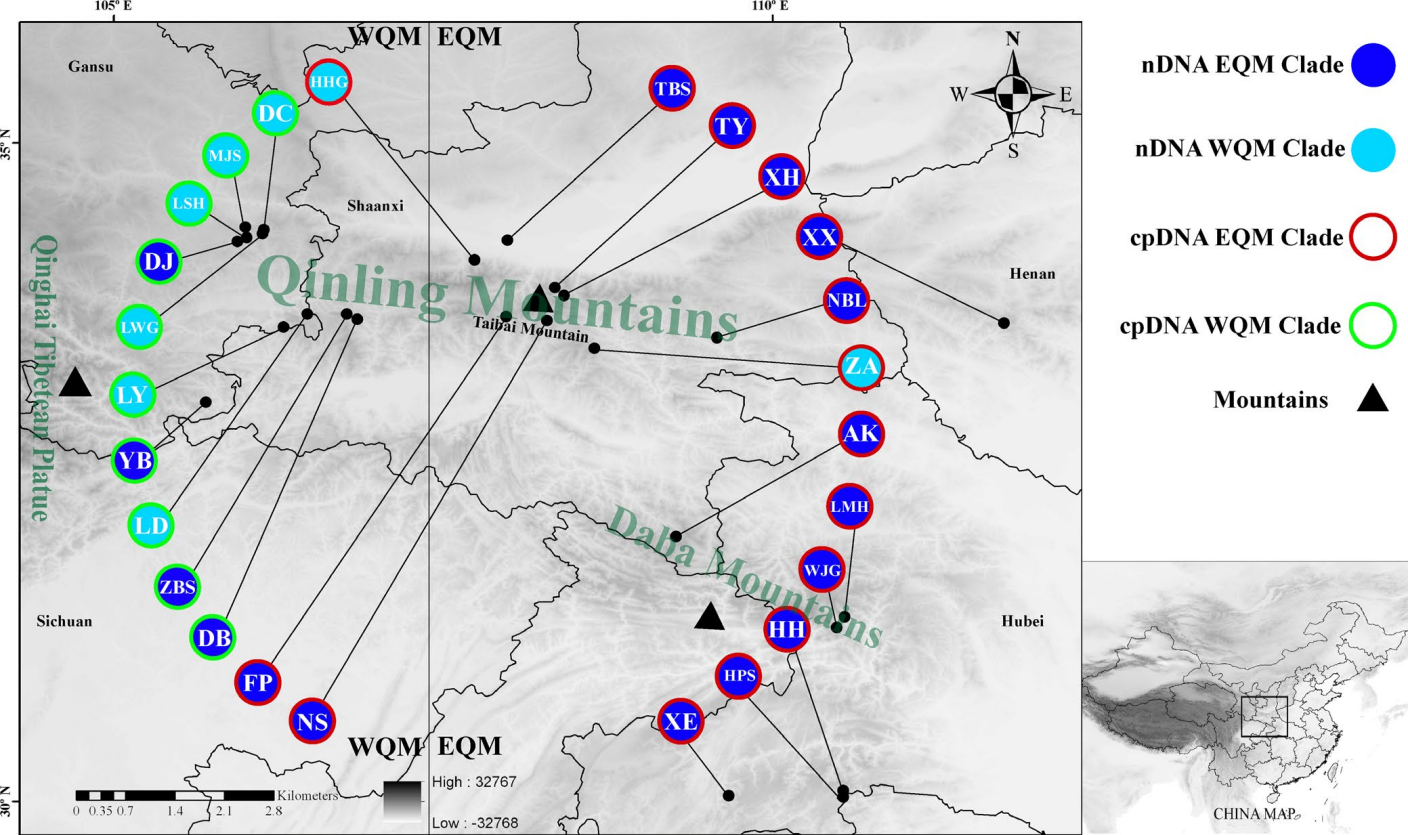
Geographic locations of all populations of*Dipteronia sinensis*in the Qinling Mountains area. Each circle represents a population sample. The two distinct colors for circles represent the two clades identified based on the nDNA sequences (SNP loci). The two different outlines for circles represent the two clades identified based on the whole chloroplast genomes. “WQM and EQM” represent the west and east sides of the Qinling Mountains, respectively

**FIGURE 2:**
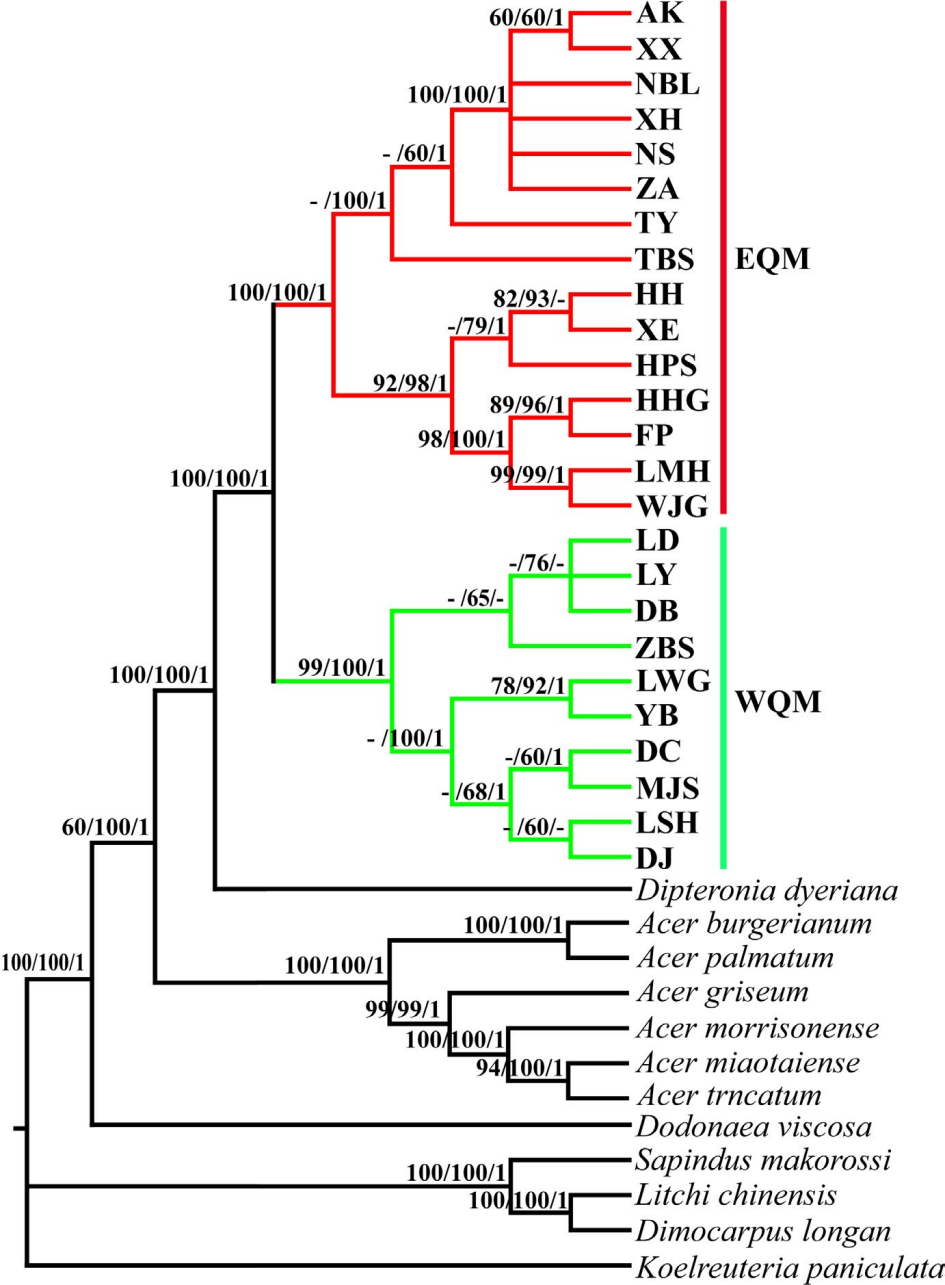
Phylogenetic trees obtained for*Dipteronia sinensis*based on the whole chloroplast genome sequences. Red and green indicate the populations from the east and west sides of the Qinling Mountains denoted as “EQM and WQM,” respectively. Bootstrap support values are shown above the branches (>50% in each case). The first bootstrap value represents that for maximum likelihood (ML), the middle value represents that for maximum parsimony (MP), and the last value represents that for Bayesian inference (BI)

**FIGURE 3:**
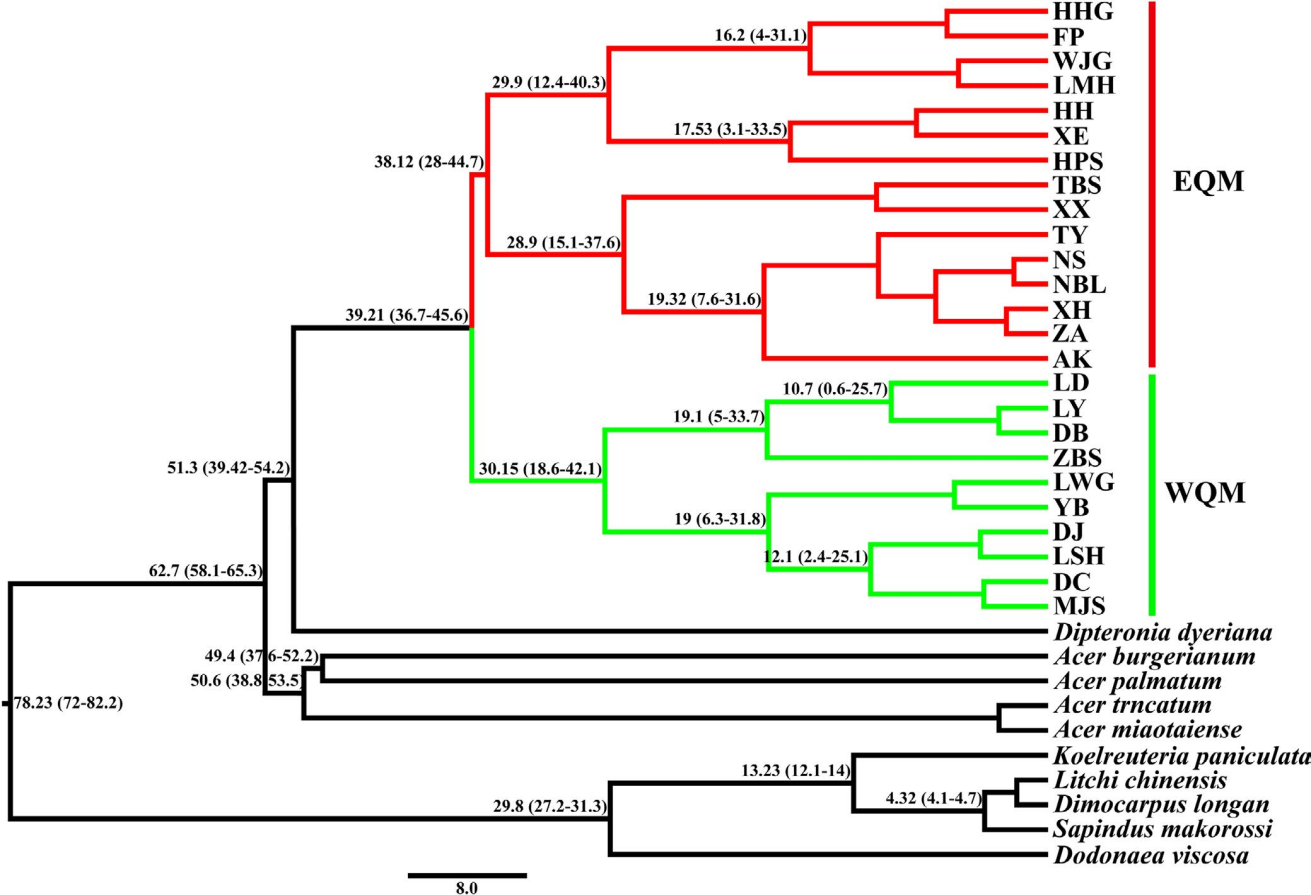
Chronogram obtained for*Dipteronia sinensis*based on the whole chloroplast genome sequences using BEAST. Red and green indicate the populations from the east and west sides of the Qinling Mountains denoted as “EQM and WQM,” respectively. The mean divergence time and 95% HPDs are labeled above the line in Mya

Phylogenetic analysis based on the complete chloroplast genome data set obtained a similar topology using the three methods employed (ML, MP, and BI cladograms) (Figure 2). The phylogenetic tree formed a larger bi‐phyletic clade with high bootstrap support values, but two clades clearly diverged between the eastern and western sides of the QM (EQM and WQM, respectively) (Figures 1 and 2). An identical topology was obtained with the highest posterior probability of 1.0 by BI analysis. We also estimated the divergence times for the intraspecific *Dipteronia sinensis* lineages based on the cpDNA data. Our molecular dating results suggested that the species *D. sinensis* diverged from its relatives during the early Paleogene at 62.7 Mya (58.1–65.3 Mya, 95% highest posterior density (HPD)) and from its sister species *D. dyeriana* about 51.3 Mya (39.42–54.2 Mya, 95% HPD). The first divergence time for *D. sinensis* into the EQM and WQM was estimated at 39.21 Mya (36.7–45.6 Mya, 95% HPD) in the late Eocene era. The EQM lineage populations diverged about 38.12 Mya (28–44.7 Mya, 95% HPD), and the estimated divergence time for the WQM lineage populations was 30.2 Mya (18.6–42.1 Mya, 95% HPD) (Figure 3).

We sequenced 23 *Dipteronia sinensis* populations by genotyping to produce a maximum of >744 million reads for each population. The estimated percentage of GC contents ranged from 41.47% to 39.05%. In total, 28,246 SNP loci were generated for further population genetics analyses. Phylogenetic analysis based on the nDNA SNPs identified three *D. sinensis* clades, where two clades contained most of the EQM populations and WQM populations. However, one clade contained a mixture of the EQM and WQM populations (Figure 6).

### 3.2 STRUCTURE analyses

**FIGURE 4:**
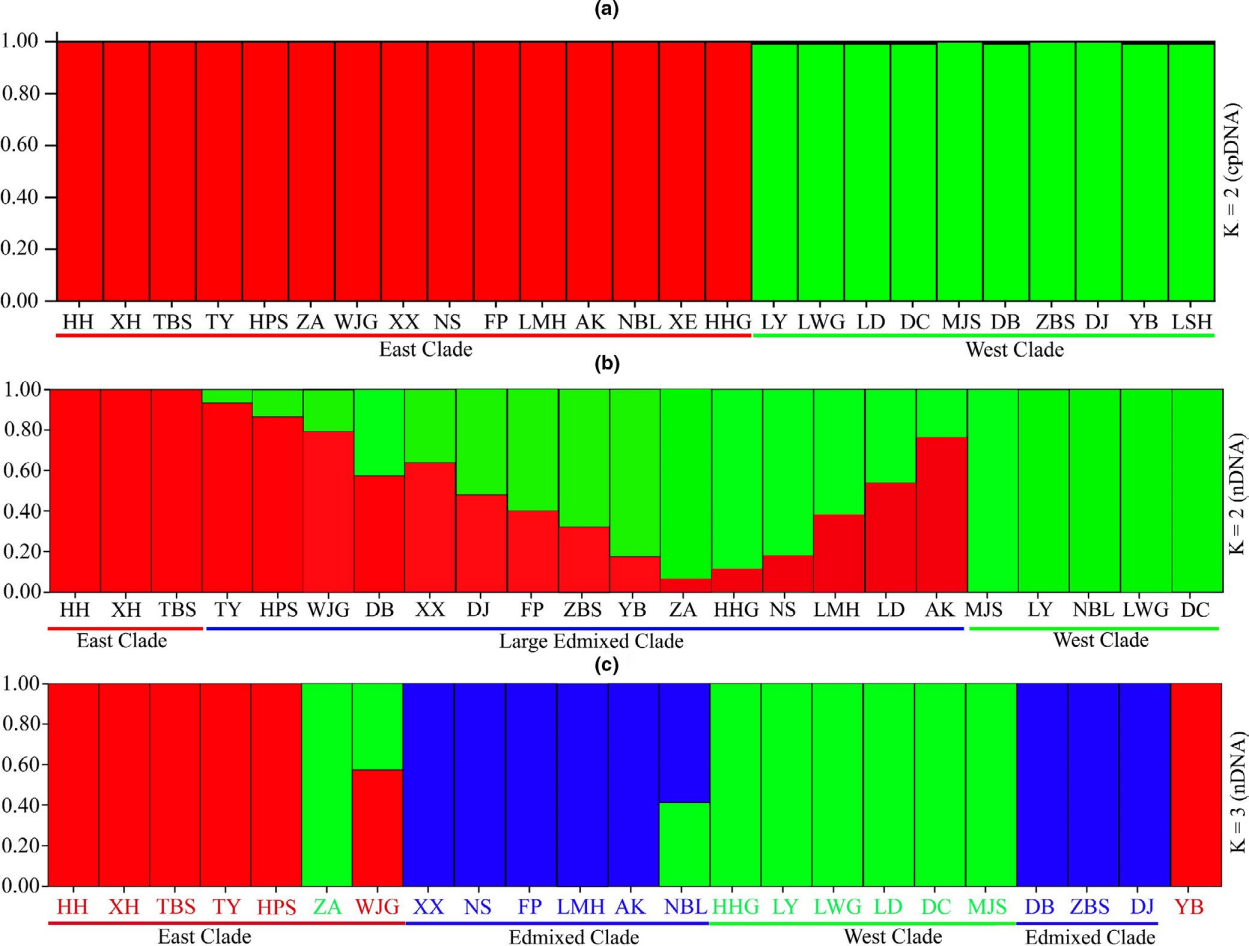
STRUCTURE results obtained for*Dipteronia sinensis*based on: (a)*K* = 2 whole chloroplast genome (cpDNA) data, where red and green represent the populations from the east and west sides of the Qinling Mountains, respectively, as identified in the phylogenetic tree: (b)*K* = 2 nuclear DNA (SNP loci) sequences, where the results are shown as three clades, with red and green representing the populations from the east and west of the Qinling Mountains, and a large admixed clade is shown in both colors and: (c) nuclear DNA (SNP loci) sequences, where the results are shown as three clades, with red, green, and blue representing the populations from the east and west of the Qinling Mountains, and the mixed population,*K* = 3, respectively. The x‐axis indicates the population names, and the*y*‐axis shows the STRUCTURE values

The results obtained by STRUCTURE analyses based on the cpDNA variations were the same as those produced using the phylogenetic tree. They suggested the clear intraspecific divergence of *Dipteronia sinensis* into EQM and WQM lineages (Figures 2 and 4a). However, no gene flow was detected between the EQM and WQM lineage populations (Figure 4a).

**FIGURE 5:**
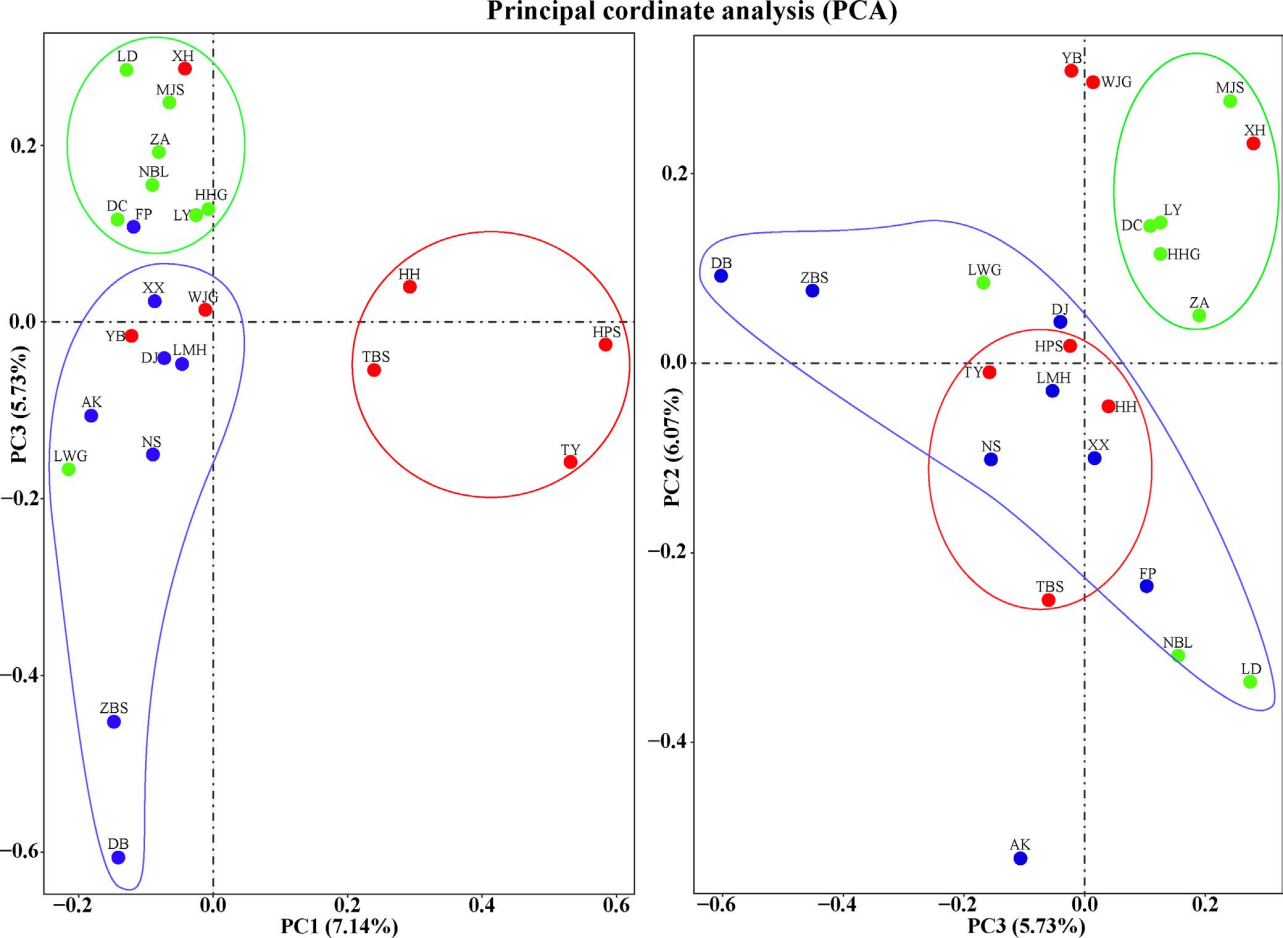
Principal component analysis results obtained for*Dipteronia sinensis*based on SNPs (nDNA) data. Red, green, and blue dots represent the east, west, and mixed populations, respectively, and red, green, and blue circles represent the east, west, and mixed population groups, respectively. Each axis shows the percentage of genetic variation in brackets

**FIGURE 6:**
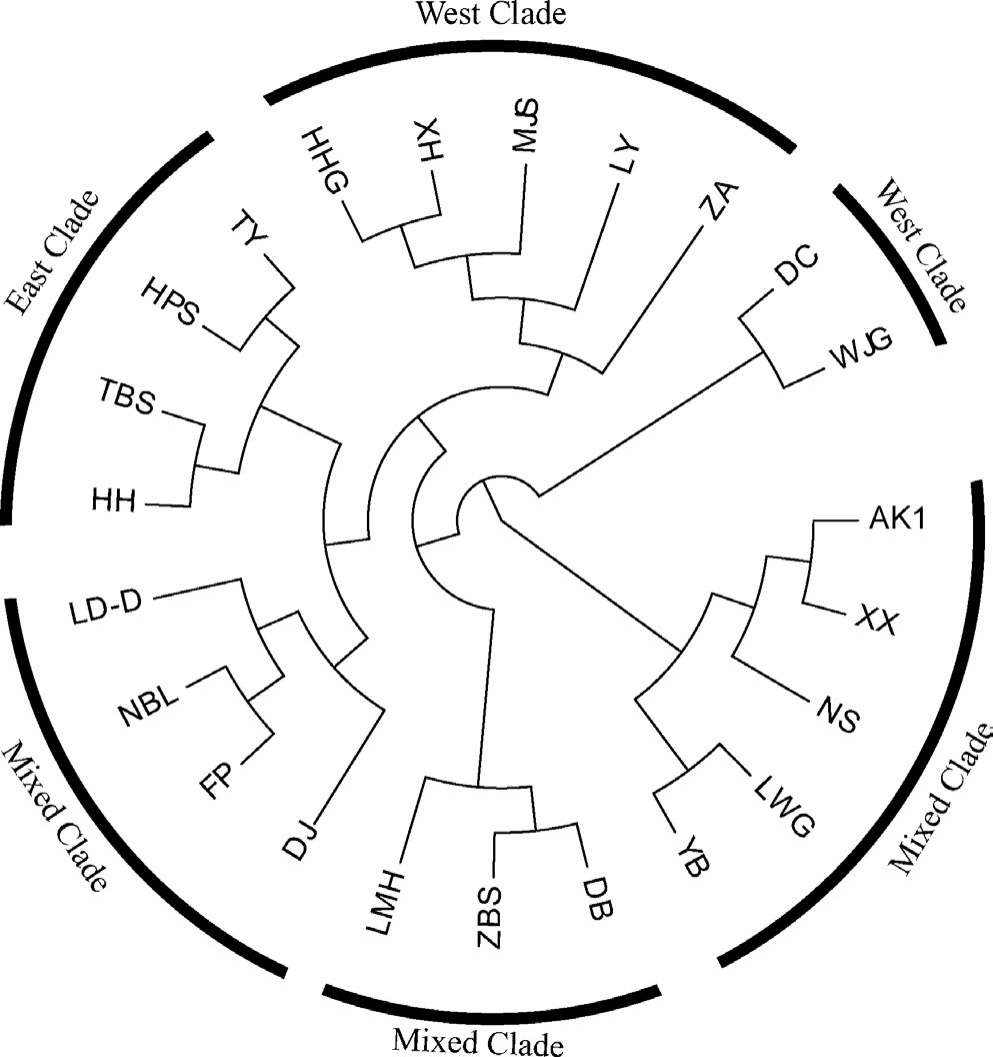
Phylogenetic relationships obtained for 23 populations based on SNP data

STRUCTURE analysis (*K* = 3) based on nDNA SNP data obtained a different genetic structure than that with the cpDNA results (Figure 4c). Our nDNA results detected three groups of populations, where two groups diverged into the EQM lineage (HH, HX, TBS, TY, HPS, WJG, and YB) and WQM lineage (ZA, NBL, HHG, LY, LWG, LD, DC, and MJS). Another group of populations called the “mixed populations clade” (Figure 4c) contained a mixture of the populations from both sides (EQM and WQM). Moreover, our PCA results (Figure 5) and phylogenetic tree (Figure 6) based on nDNA also detected three major populations groups, where two groups were clearly the EQM and WQM populations, and the other was a mixture of both sides.

### 3.3 Demographic dynamics

We calculated the nucleotide diversity Pi (*π*) and Theta (*θ*) by using DnaSP software based on the SNP loci (cpDNA). We estimated that the nucleotide diversity was much higher for the EQM lineage (*π* = 0.00026 and *θ* = 0.00031) than the WQM lineage (*π* = 0.00009 and *θ* = 0.00011).

IMa software was used to analyze the gene flow migration between the EQM and WQM lineages. The plot obtained contained a sharp peak for the posterior density (results not shown). The mean effective population size estimated for the EQM lineage (q1 = 364.89, 95% HPD 913.48–201.72) was much higher than that for the WQM lineage (q2 = 81.25 95% HPD 286.81–39.88) (Table 3). The daughter populations (q1 and q2) were larger than the ancestral population (qA = 134.31), thereby indicating that the EQM lineage might have experienced population expansion. The gene flows from EQM to WQM (m1) and from WQM to EQM (m2) were estimated as the same (m1 = 0.0050; m2 = 0.0050, *p* < .001).

**TABLE 3:** Isolation‐with‐migration analysis of *Dipteronia sinensis* based on single nucleotide polymorphism sequences (cpDNA)

Species	Level	q1	q2	qA	m1	m2	T (generations)
*Dipteronia sinensis*	Hipt	364.89	81.25	134.31	0.0050	0.0050	22.01
	95% HPD (High)	913.48	286.81	654.61	8.30	8.11	29.51
	95% HPD (low)	201.72	39.88	65.90	0.0050	0.0050	6.77

Note

q1, Effective population size per individual from the east side to the west side; q2, effective population size per individual from the west side to the east side; qA, ancestor's population rate per individual; m1, migration of gene flow from the east side to the west side; m2, migration of gene flow from the west side to the east side; *t*, divergence time.

PCA (PC1, PC2, and PC3) identified the same genetic pattern as STRUCTURE analysis based on the nDNA data. Three different genetic groups were determined by PCA, which contained the EQM and WQM populations, and a mixture of the populations from both sides, as shown in Figure 5. The three axes comprising PC1, PC2, and PC3 estimated very low genetic variation among each group, that is, 7.14%, 6.07%, and 5.73%, respectively (Figure 5). The PC1 geographic axis indicated the high correspondence between the genetic data for the EQM and WQM population groups. Figure 5 shows that the EQM populations (TY, TBS, HPS, and HH) and WQM populations (LD, MJS, ZA, NBL, HHG, LY, and DC) were on the two extreme sides of the distribution map. By contrast, the remaining populations were isolated from the groups and formed a “scattered populations group” on the PC1 axis. The geographic distance on the PC3 axis was a substructure of PC1 representing the increase in the geographic range from the EQM to WQM population groups (Figure 5). The large gap between the two groups of populations suggested a significant difference in those genetic heredity groups. Ideally, individuals with a similar genetic background will be clustered together by PCA.

## 4 DISCUSSION

### 4.1 Intraspecific divergence of *D. sinensis*

We examined the divergence of *Dipteronia sinensis* lineages based on the complete chloroplast genomes (cpDNA) and large‐scale SNP variations in the QM and adjacent areas. Phylogenetic analysis based on cpDNA clearly determined the bi‐phyletic clades within the species (EQM versus. WQM) (Figures 1 and 2). The present‐day abutting ranges of *D. sinensis* in EQM to WQM reflect this deep divergence. Therefore, we estimated the divergence times for the intraspecific *D. sinensis* lineages as occurring in the late Eocene at about 39.2 Mya (95% HPD, 36.7–45.6 Mya) (Figure 3). Phylogenetic analysis also showed that the divergence of two species in the genus *Dipteronia* occurred at about 51.3 Mya (39.42–54.2 Mya, 95% HPD), which is consistent with a previous phylogenetic analysis based on transcriptome data sets (Feng et al., 2018). Therefore, our phylogenomic dating strongly suggests that *D. sinensis* and *D. dyeriana* qualify as “living fossils” that have experienced long‐term morphological stasis, possibly since the Paleocene/Eocene (Figure 3). They survived the late Tertiary/Quaternary periods with adverse climatic conditions in subtropical China's mountainous areas (Qiu et al., 2011).

The orogenesis of the QM was estimated to have begun in the Late Triassic (~230 Mya) (Zhang et al., 1996). However, the current topographical features of the QM mainly formed starting with a small‐magnitude uplift at 40–70 Mya and a rapid large‐magnitude uplift beginning at 1.2–2.4 Mya (or 0.7 Mya), especially for the higher mountain features of the Taibai Mountains (Dong et al., 2011; Liu et al., 2010; Ying, 1994). Given that the main phylogeographical patterns occur along the west–east axis, we suggest that historical isolation due to the QM's small‐magnitude orogenesis during the Early Cenozoic (60–35 Mya) might have caused the genetic divergence between the two Qinling lineages of *Dipteronia sinensis*. Thus, the small and rapid large magnitude of QM collectively during the Cenozoic has led to complementary evolutionary and ecological isolation, and the high richness of endemic species. Our divergence estimates based on the cpDNA results suggest that the small‐magnitude uplift (early Paleocene to late Eocene) of the QM might have been an important factor responsible for the genetic alteration of *D. sinensis*. Recently, the divergence time of the genus *Sinothela* was estimated at between 23–2.6 Mya or 10–2.6 Mya in the QM (Xu et al., 2018). Some recent phylogeographical studies have also demonstrated that species distributed at high altitudes diverged due to geological changes and Tertiary climatic oscillations in the west–east and west–central–east parts of the QM (Fang et al., 2015; Wang et al., 2013; Yang et al., 2015). Our results suggest that the uplift of the QM and consistent climatic blockage from the mountain peaks may have affected the genetic divergence of alpine plants.

Interestingly, the maternally inherited cpDNA data supported the intraspecific divergence of *Dipteronia sinensis*, whereas little congruence was found between the genome‐wide SNP data and cpDNA markers. In general, these two sets of markers are very useful for tracing genetic variations, lineage divergence, and the responses of high alpine plants in terms of their ranges to climatic oscillations (Wang et al., 2009). Our PCA and STRUCTURE analysis results based on nDNA showed that some populations (e.g., XX, DB, FP, LMH, NS, ZBS, and AK) formed a different clade called the “mixed populations clade” (Figures 4c and 5). However, the populations distributed in the WQM and EQM clades were the same as those based on the cpDNA markers. Thus, why did the nDNA (SNP) data yield an admixture of some populations from the WQM and EQM lineages, whereas the cpDNA data produced a clear genetic structure? The inconsistency between the results obtained based on nuclear DNA (SNPs) and the complete chloroplast genome variations was possibly due to the rate of lineage sorting and gene flow by the two markers. In general, the cpDNA data indicated a smaller effect on the population size due to low mutation and evolutionary rates compared with the nDNA data. By contrast, the nDNA data revealed a more rapid evolutionary rate and larger significant population size than the cpDNA data (Wolfe et al., 1987). Therefore, the rate of lineage sorting was more rapid for cpDNA than nDNA (Toumi & Lumaret, 2010).

Moreover, the low evolutionary rate of the cpDNA markers restricted the gene flow (Philippe et al., 2011), and the fixed lineage sorting of the ancestral polymorphisms may have contributed to the incongruence between the results obtained based on cpDNA and nDNA data (Wang et al., 2014). We found that when *K* = 2, the STRUCTURE results (*K* = 2) based on nDNA SNP data comprised two large admixed population groups (Figure 4b). However, when *K* = 3, a different genetic structure was obtained based on the SNPs data, as shown in Figure 4c. The results demonstrated that among the three groups of populations, two groups diverged into the EQM lineage (HH, HX, TBS, TY, HPS, WJG, and YB) and WQM lineage (ZA, NBL, HHG, LY, LWG, LD, DC, and MJS), but the third group contained a mixture of the populations from both sides (EQM and WQM), possibly due to the different rates of lineage sorting and the evolutionary rates of the two markers. The genetic compositions of the ZA and YB populations were also the opposite based on the chloroplast loci and nDNA SNP data because the long‐distance dispersal of pollen blurred the genetic structure according to the cpDNA variations. Similarly, some Mediterranean and East Asian species exhibit introgression between cpDNA and nDNA (Meng, 2017; Vitelli et al., 2017). Another factor related to the incongruent results is nDNA incompatibility, as described by Abe's rules of nDNA and cpDNA dispersal (as in Haldane's rule), or sex differences (Abe et al., 2005; Di‐Candia & Routman, 2007). For example, cpDNA is maternally inherited (e.g., seeds) (Bartish et al., 2002) and nDNA is biparentally inherited (e.g., seeds and pollens) (Sun et al., 2002).

### 4.2 Demographic history

In the present study, STRUCTURE analysis obtained a clear genetic pattern for *Dipteronia sinensis,* and the results indicated no gene flow between two lineages in the EQM and WQM areas. The results obtained by IM analysis (with IMa) also suggested little gene flow between the two lineages. Previously, it was reported that divergence due to geographical tectonics or climatic isolation could produce a strong signature because of highly restricted gene flow (Avise, 2012; Parchman et al., 2006). Several studies have suggested that climatic change during the ice ages hindered the gene flow among populations, thereby resulting in the divergence of species lineages and the formation of new population lineages/taxa (Jiang et al., 2007; Ye et al., 2016). Studies have also shown that the gene flow might have been limited due to the QM forming a robust geographical barrier between northern and southern China (Fang et al., 2015; Yuan et al., 2012; Zhang et al., 2014).

Similarly, evidence indicates that the formation of the Yangtze and Yellow Rivers due to the QM uplift during the Pliocene affected the genetic structure and limited the gene flow (Duan et al., 2009; Zhao et al., 2011). A study based on *Paeonia rockii* showed that the gene flow was limited between populations due to geographic isolation in the QM area (Yuan et al., 2010, 2011). Thus, these results suggest that tectonic changes and climatic adaptation during the Pliocene might have counteracted interspecific gene flow, thereby promoting the divergence of lineages (Feder et al., 2012).

Interestingly, our IMa estimates indicated that the daughter populations (q1) of *Dipteronia sinensis* were much larger than those of the ancestral population (qA) (Table 3), which suggests that the *D. sinensis* populations expanded. Similarly, other studies of plants indicate that the ranges of species expanded during glacial periods where species survived in microrefugia (cryptic refugia) (Wang et al., 2009). During the Pleistocene, the climatic oscillations affected the genetic architectures of most species and caused range expansions in regions with climatic changes (Avise, 2000; Hewitt, 2001). Therefore, our IM results support the hypothesis that the Pleistocene climatic oscillations were related to genetic variation and the expansion of *D. sinensis* in the QM and adjacent areas (Table 3). Previously, it was reported that climatic changes could drive range expansions of species (Avise, 2000) to promote phenological radiation and the generation of various morphotypes or species diversification (Milá et al., 2007). Phylogeographical studies suggest that populations of alpine species exhibited evident range retreat patterns and expansion during the Pleistocene climate oscillations (Zhang et al., 2005). Based on the discussion above, we may conclude that the divergence and expansion of species populations triggered by Pleistocene changes promoted species differentiation in plants.

### 4.3 Conservation strategies for *D. sinensis*

The ecological and evolutionary units that maintain biodiversity should be protected and conserved. *Dipteronia sinensis* is a naturally endangered and relict tree species included in the Red List, and thus, there is a critical need to design effective conservation strategies for this plant (Cowling & Pressey, 2001; Davis et al., 2008). This is the first study to provide details of the evolutionary history and genetic structure of *D. sinensis* based on cpDNA and nDNA SNP data. In addition, our analysis of the demographic history of *D. sinensis* indicated low nucleotide diversity and restricted gene flow between the WQM and EQM clades of the widely distributed populations of *D. sinensis*, thereby supporting our conclusion that the WQM and EQM populations are not interbreeding (Figure 4a). However, during field investigations, we found that illegal human activities (e.g., continuous harvesting, development of agriculture, the rise of tourism activities, low reproductive capacity, hydroelectric development, and other industrial actions) and genetic fragmentation have significantly depleted several natural populations of *D. sinensis* (Hamilton et al., 2012; Zhou et al., 2010). Due to the continuous decreases in the sizes of wild populations, it is necessary to protect the populations affected by human activities in order to save the natural ecosystem. The direct effects of climate change are even more significant on alpine ecosystems, and they will be a much greater challenge for nature conservation in the 21st century. Details of factors such as topographic features, climatic changes, the growth of alpine ecology, and the population genetic structure and phylogeography of *D. sinensis* are necessary to facilitate the conservation of valuable plant habitat resources (Schmitt, 2007). Therefore, two different conservation and management units should be constructed for *D. sinensis* in the EQM and WQM areas. In addition, the conservation of natural populations, habitat diversity, and enhanced gene exchange among populations in different locations can be improved by collecting mature seeds from various populations and artificially planting them in botanical gardens (Cabrera‐Toledo et al., 2012).

## CONFLICT OF INTEREST

The authors have no conflicts of interest to declare.

## AUTHOR CONTRIBUTION

**Khurram Shahzad:** Formal analysis (lead); Methodology (lead); Project administration (equal); Software (lead); Writing‐original draft (lead); Writing‐review & editing (equal). **Mi‐Li Liu:** Methodology (equal); Writing‐review & editing (equal). **Yu‐He Zhao:** Methodology (equal); Software (equal); Writing‐review & editing (equal). **Ting‐Ting Zhang:** Methodology (equal); Writing‐review & editing (equal). **Jian‐Ni Liu:** Data curation (equal); Supervision (equal); Writing‐review & editing (equal). **Zhonghu Li:** Project administration (equal); Software (equal); Supervision (equal); Writing‐original draft (equal); Writing‐review & editing (equal).

## Data Availability

DNA sequences: GenBank accession numbers NCBI: MK193760–MK193784. Final DNA sequence annotations are uploaded online as above.
